# The 2020 American College of Cardiology/American Heart Association (ACC/AHA) Guideline for the Management of Patients with Valvular Heart Disease. Should the World Jump In?

**DOI:** 10.21470/1678-9741-2021-0953

**Published:** 2021

**Authors:** Walter J Gomes, Rui M S Almeida, Orlando Petrucci, Manuel J. Antunes, Luciano C. Albuquerque

**Affiliations:** 1 Cardiovascular Surgery Discipline and São Paulo Hospital. Escola Paulista de Medicina. Federal University of São Paulo, São Paulo, Brazil.; 2 Faculty of Medicine of the University Center Assis Gurgacz, Cascavel, PR, Brazil.; 3 Faculty of Medical Sciences. State University of Campinas - UNICAMP, Campinas, SP, Brazil.; 4 Faculty of Medicine, University of Coimbra, Coimbra, Portugal.; 5 São Lucas Hospital of the Pontifical Catholic University of Porto Alegre, Porto Alegre, RS, Brazil.

The publication of the 2020 ACC/AHA guidelines for the management of patients with heart valve disease, while updating essential steps for clinical evaluation and diagnosis, and assessing the impact of new data, faces both methodological issues and conflict of interest, downplaying a crescendo body of evidence for adverse outcomes associated with transcatheter therapies, aggravated by the manifest conflict of interest of the writing committee authors and reviewers included in the final version of the document^[1]^. As openly stated in the document, the focus is on medical practice in the United States, but further intentioned to patients throughout the world and may be used to inform regulatory or payer decisions.

The market-driven health system in the U.S. has been generating tremendous influence on scientific evidence, where for-profit purposes have been confronting aspects on good medical practice and patient safety. This includes an extensive part of randomized controlled trials (RCT) with funding aimed at favoring costly and often unnecessary procedures, with premature and distorted conclusions, and with long-term results still pending. Therefore, the ACC/AHA document seems disconnected from the medical and health care reality of most countries, especially the emerging and underdeveloped countries, conflicting even with the reality of the health system in the United States of America.

While the United States delivers some of the most technologically advanced medicine and is a medical research leader, according to a report from the The Commonwealth Fund, the United States has spent more on healthcare than any high-income country. In 2018, the expenditure on healthcare totaled 17.7% of its Gross Domestic Product - a whopping 3.6 trillion dollars - and among 11 countries from the Organization for Economic Co-operation and Development’s (OECD) analyzed and compared, the U.S. ranked last place for health outcomes, equity, and quality, despite having the highest per capita health earnings. It leaves 10.9% of the United States population deprived of access to health care (more than 30 million people in 2019), and 31 million more are underinsured, together comprising approximately 40% of adults under the age of 65^[2,3]^.

Meanwhile, a report from the World Bank and World Health Organization discloses that half the world population (more than 3 billion people) lacks access to essential health services, and 100 million still pushed into extreme poverty because of health expenses, forcing them to survive on just $1.90 or less a day, making necessary a fundamental shift in the way resources are mobilized for health and human capital, especially at the country level. And the COVID-19 pandemic may have made these figures even worse^[4]^.

Approximately 50% of authors and reviewers and/or their institutions disclose a conflict of interest, although the vote of authors with a conflict of interest in related matters has been avoided, the influence on recommendations is a lingering issue. The main evidence used for recommendations, mainly RCTs, presents funding and industry interference at all stages, several with important gaps, inference bias, and premature conclusions.

The ACC/AHA document fails to examine important existing evidence and to make recommendations in line with the goals of treating heart valve disease. The treatment of aortic stenosis (AS) should be performed to restore long-term survival and improve quality of life, avoiding harmful late events that reverse the success of the procedure. While conventional surgery and transcatheter therapy are presented as options, for now only surgical aortic valve replacement (SAVR) is assumed to restore the prognosis of patients with symptomatic severe AS, with postoperative long-term survival becoming comparable to an age- and sex-matched general population without AS^[5-8]^.

Although open surgery has been the gold standard for the treatment of severe AS for decades, the introduction of transcatheter aortic valve implantation (TAVI) established a new paradigm, less invasive and with early hospital discharge rather than the need for sternotomy and longer hospital stay with SAVR.

## Emerging Evidence

Interim conclusions drawn from RCTs are being contradicted by many long-term follow-up and analysis of registries and national databases with the inclusion of a large number of patients and real-world data. While the ACC/AHA document reinforces that the goal of valve intervention is to improve symptoms, prolong survival and minimize the risk of related complications, it overlooks the evidence already available when pondering the impact of different therapies on long-term survival, with recommendations purportedly anticipating similar outcomes. These issues become critical at a time when TAVI has been indicated to younger and lower-risk groups of patients with AS and, more concerning, the drive for expanding the device utilization with earlier intervention in asymptomatic patients with AS^[9,10]^.

In patients with low to intermediate surgical risk, TAVI has demonstrated a clinical effect and survival equivalent to SAVR in the 2-year follow-up. However, in the extended follow-up, the evidence continually emerging shows elevated complication rates with higher long-term mortality with TAVI compared to surgical treatment^[11]^. In this way, the report of the five-year outcomes of PARTNER 2 cohort A trial found a higher risk of death or disabling stroke between 2 and 5 years after TAVI than after SAVR, with a hazard 27% higher, stirring concerns regarding the long-term effectiveness of TAVI^[11,12]^.

A meta-analysis by Barili et al.^[13]^ with Kaplan-Meier-estimated individual patient data evaluating the effects of TAVI and SAVR on the long-term all-cause mortality rate revealed a lower incidence of death in the first year after TAVI [risk-profile stratified hazard ratio (HR) 0.85, 95% confidence interval (CI) 0.73-0.99;*P*=0.04], whereas there was a reversal of the HR after 40 months (risk-profile stratified HR 1.31, 95% CI 1.01-1.68;*P*=0.04) favoring SAVR over TAVI. The mortality rates in trials of TAVI *versus* SAVR are affected by treatments with a time-varying effect and TAVI is related to better survival in the first months after implantation whereas, after 40 months, it is a risk factor for all-cause mortality^[13]^.

A meta-analysis from Takagi et al.^[14]^ evaluating mortality with ≥5 years of follow-up in RCTs and propensity-score matched (PSM) studies of TAVI *versus* SAVR included 3 RCT and 7 PSM enrolling 5498 patients. The pooled analysis of all 10 studies demonstrated a statistically significant 38% increase in mortality with ≥5-year follow-up with TAVI relative to SAVR. A subgroup meta-analysis showed no statistically significant difference between TAVI and AVR in RCTs and a statistically significant 68% higher with TAVI relative to SAVR in PSM studies^[14]^.

Wang et al.^[15]^, assuming that results from RCTs and real-world study (RWS) appear to be discordant, investigated whether data derived from RCTs and RWS evaluating long-term all-cause mortality of TAVI *versus* SAVR were in agreement. Five RCTs (5421 participants, TAVI: 2759, SAVR: 2662) and 33 RWS (20839 participants; TAVI: 6585, SAVR: 14254) reporting long-term (≥2-year follow-up) all-cause mortality were identified. Pooled RCT analysis showed no difference in all-cause mortality between TAVI and SAVR (HR=0.97, 95% CI: 0.88-1.07; *P*=0.55). In RWS, TAVI was associated with an increased risk of all-cause mortality (HR=1.46, 95% CI: 1.26-1.69; *P*<0.001) compared to SAVR, highlighting the inconsistencies between RCTs and RWS in assessing long-term all-cause mortality in the treatment of AS using TAVI or SAVR^[15]^.

In the Karlsruhe registry, the propensity score-matched analysis of patients who underwent TAVI (n=216) and SAVR (n=216) between 2008 and 2012 showed that TAVI patients had lower survival rates at 6 years than SAVR patients (40.7% *vs*. 59.6%, respectively, *P*<0.001, HR 2.15; 95% CI 1.45 to 3.20)^[16]^.

Sayed et al.^[17]^ compared in a meta-analysis the outcomes of TAVI and minimally invasive aortic valve replacement in the management of aortic stenosis (AS). Including a total of 11 cohort studies, of which seven were matched/propensity-matched, demonstrated higher rates of midterm mortality (≥1 year) with TAVI (HR: 1.93, 95% CI: 1.16 to 3.22), but no significant differences with respect to 1-month mortality (HR: 1.00, 95% CI: 0.55 to 1.81)^[17]^.

In the PARTNER-3 trial, the event-rate lines for death and disabling strokes, which significantly favored TAVI in the 1-year analysis, from the 2-year follow-up the curves are converging over time, and reversal of fortunes may become tangible in the longer-term^[18]^.

This emerging data reveals that the rising long-term mortality is a consequence of aggregated specific complications occurring during and after the TAVI procedure.

## Paravalvular regurgitation

Paravalvular regurgitation (PVR), particularly when moderate or severe, is recognized as a significant complication occurring after TAVI, with an incidence ranging from 3.5% to 12%, and is associated with a 3-fold increase in mortality in 30 days and 2.3 times in 1-year, in addition to increasing rehospitalization for heart failure^[12,16,19-20]^. However, the late prognosis of mild PVR was less clear, as the incidence is higher and can vary from 20% to 40%, depending on the type of prosthesis and the characteristics of the patient’s population. In the intermediate-risk patients in the PARTNER 2 trial, the rate of mild PVR at 30 days was 22.5% after TAVI and 2.8% after SVR, while the rate of moderate to severe PVR was 3.7% after TAVI and 0.6% after SAVR^[21]^.

Refinements in device design and technique have considerably reduced the incidence of PVR. However, at 30 days, there is still a higher rate of moderate and severe PVR with TAVI compared to SAVR (0.8% *versus* 0.0% in PARTNER 3 and 3.5% *versus* 0.5% in Evolut Low-Risk trials^[18,22]^. The most recent modifications of TAVI systems designed to minimize PVR seem to result in greater direct trauma to the conduction system, resulting in a higher incidence of left bundle branch block (LBBB) and raised need for permanent pacemaker implantation (PPI)^[18]^. In the PARTNER trial, the rate of mild aortic PVR was 38% and associated with increased mortality at 1 year when compared to the group with no- or trivial PVR^[20]^.

Meta-analysis by Ando et al.^[23]^ demonstrated higher all-cause mortality in patients with mild PVR compared to none/trivial PVR (HR 1.26, 95%CI 1.11-1.43, I2 =45%, *P*<0.001), with follow up ranging from 6 months to 5 years^[23]^. These findings were reinforced by a recent publication from the Finnish national registry - FinnValve - gathering data from 6463 consecutive patients who underwent TAVI (n=2130) or SAVR (n=4333) during 2008-2017 and investigating the impact of PVR at discharge on 4-year mortality. The rate of mild PVR was 21.7% after TAVI *vs*. 5.2% after SAVR and moderate-to-severe was 3.7% *vs*. 0.7%, respectively. After TAVI, 4-year survival was 69.0% in patients with none-to-trace PVR, 54.2% with mild PVR [(HR) 1.64, 95% confidence interval (CI) 1.35-1.99] and 48.9% with moderate-to-severe PVR (adjusted HR 1.61, 95% CI 1.10-2.35). After SAVR, mild PVR (4-year survival 78.9%; adjusted HR 1.29, 95% CI 0.93-1.78) and moderate-to-severe PVR (4-year survival 67.8%; adjusted HR 1.36, 95% CI 0.72-2.58) were associated with worse 4-year survival compared to none-to-trace PVR (4-year survival 83.7%), but the difference failed to reach statistical significance. Therefore, mild and moderate-to-severe PVR were independent predictors of worse survival after TAVI. Mild and moderate-to-severe PVR are infrequent after SAVR but tend to decrease survival also in these patients^[24]^.

The 2020 ACC/AHA Guideline for the Management of Patients with Valvular Heart Disease stop short of making recommendations to address the management of a patient affected by this not infrequent and potentially deadly post-procedural complication.

## Conduction disturbances

In comparison with SAVR, TAVI is associated with a 3-fold higher incidence of left bundle branch block (LBBB), as well as an increased incidence of need for a permanent pacemaker. The average rates of postoperative permanent pacemaker implantation (PPI) after TAVI have been reported ranging between 15-33%, with a significant difference according to the type of TAVI device used (20-22). In the GARY registry, the incidence was 23.7% in transfemoral TAVI^[22,23]^, while the incidence after SAVR is reported around 3%^[25,26]^.

The clinical impact of new-onset persistent LBBB (NOP-LBBB) and PPI after TAVI was evaluated in a meta-analysis including 30 trials and 59,719 patients. NOP-LBBB was associated with an increased risk of all-cause death, cardiac death, heart failure hospitalization, and PPI at 1-year follow-up. Periprocedural PPI after TAVI was associated with a higher risk of all-cause death and heart failure hospitalization. NOP-LBBB and PPI after TAVI are associated with an increased risk of all-cause death and heart failure hospitalization at 1-year follow-up. Periprocedural NOP-LBBB also increased the risk of cardiac death and PPI within the year following the procedure^[27]^.

In another meta-analysis of 17,139 patients from 40 studies, the rate of implantation of new pacemakers ranged from 16% to 37% with CoreValve; from 14.7% to 26.7% with Evolut-R; from 2.3% to 28.2% with Sapien/XT; and from 4% to 24% with Sapien 3^[28]^. The finding was correlated with longer hospital stay, left ventricular dysfunction, readmission, cost of the procedure, and late mortality^[29,30]^.

In the PARTNER 3 trial, the incidence of a new LBBB at 1 year after implantation of Sapien Valve system was 23.7% in the TAVI group compared to 8.0% in SAVR^[31]^.

One-year results from the SOLVE-TAVI trial, a direct comparison of the self-expanding CoreValve™ Evolut™R (Medtronic) and the balloon-expandable Sapien 3 (Edwards Lifesciences) in patients with symptomatic high-risk severe AS show that although implant rates of permanent pacemakers were similar between devices, they were relatively high in both arms of the study: 24.7% with Evolut™R and 20.2% with Sapien 3^[30]^. The most recent modifications of TAVI systems designed to minimize paravalvular aortic leak seem to result in greater direct trauma to the conduction system, resulting in an even higher incidence of LBBB and the need for PPI^[33,34]^. Both right ventricular apical stimulation and new LBBB were associated with a lower ejection fraction, increased hospitalizations for heart failure, and higher mortality. The long-term implications for younger patients are worrisome and may reduce, or even invalidate the initial benefit of TAVI over SAVR.

## Stroke

The incidence of stroke after TAVI has been reported to be comparable to that of SAVR. Contemporary data including different TAVI devices in high- and intermediate-risk patients show a 30-day stroke rate ranging from 1.4% to 1.9%^[36-40]^. However, the one-year results of the SOLVE-TAVI trial, a direct comparison of the self-expanding CoreValve™ Evolut™R (Medtronic) and the balloon-expandable Sapien 3 (Edwards Lifesciences) in patients with symptomatic high-risk aortic stenosis, revealed a significantly higher stroke rate with the Sapien 3 valve: 6.9% *versus* 1.0% with CoreValve Evolut R (*P*=0.002)^[32]^.

The largest multicenter observational registry to date evaluating the incidence of late neurological events (LCVE) after TAVI revealed an increased stroke rate in the years following the procedure associated with high mortality compared to what would be expected in an age-matched population. In this multicenter study, including 3,750 consecutive patients from seven centers in Canada, France, and Spain, the in-hospital stroke rate was 2%. After a median follow-up of 2 years, 5.1% of patients had a LCVE, which was associated with a mortality rate of 29%^[35]^.

Previous estimates of stroke incidence after TAVI were often derived from clinical trials in which interventionalists, surgeons, and patients were carefully selected, and the postprocedural assessment of neurological injury did not include clinical examinations conducted by specialists (neurologists) or sensitive image screening. In studies in which neurologists were involved in assessing stroke diagnosis, the rates of clinical events were much higher (approximately 5%-17%), and studies incorporating early magnetic resonance imaging (MRI) found very high rates of clinically silent acute brain infarction (range 74% - 100%)^[36]^.

The CLEAN-TAVI trial evaluated the effectiveness of a cerebral embolic protection device on the number and volume of cerebral lesions in patients undergoing TAVI. A total of 100 patients with severe aortic stenosis were randomized to undergo TAVI with (n=50) or without (n=50) the cerebral embolic protection device. By MRI, at 2 days after TAVI, 98% of patients in both groups had new brain lesions detected on MRI. Clinical stroke occurred in 10% of patients randomized to the protective device group versus 11% in the control group. The high rate of clinical stroke in this study, compared to previous TAVI studies (rates of clinical stroke of approximately 2% -5%) is probably related to the application of early and rigorous neurological assessments^[37]^.

In the ADVANCE trial, within the first months after TAVI using the CoreValve™ half of the reported strokes occurred on the day of the procedure or the first postprocedural day and the other half between day 2 and day 30, suggesting that the risk of stroke is not limited to the procedure itself^[38]^. The incidence of perioperative stroke after TAVI is associated with a 6-fold higher risk of mortality in 30 days^[39]^.

## Silent stroke - cerebral embolism

Failure to remove the annular calcium and the forceful dilation of the stenotic aortic valve trigger calcium embolization, causing silent brain infarctions (SBI). New ischemic brain lesions were found in 74% to 100% of patients on diffusion-weighted magnetic resonance imaging (DW-MRI) after TAVI^[37,39-40]^. Although studies have shown that SBI may not be related to apparent short-term neurological symptoms, evidence point to an association with accelerated cognitive decline and strengthening of the risk of long-term dementia (most commonly Alzheimer's disease)^[39-43]^.

Cognitive decline after cardiac surgery is related to increased morbidity and mortality^[44]^, and in the general population, SBI is associated with progressive dementia, future stroke, and increased mortality^[45]^. Dedicated meta-analyses demonstrated that SBIs of small volume < 3mm are independent predictors of later stroke and mortality^[46]^. Valvular thrombosis was recently reported as a potential mechanism of subacute stroke after TAVI. This transient thrombosis of valvular leaflets is one of the mechanisms explaining subacute events and could be the reason for the observed heightened rate of new SBIs after TAVI^[47]^.

A systematic review examining the incidence of SBI after TAVI included 39 relevant studies with 2,408 patients. Of the 2,171 patients undergoing post-procedure DW-MRI, 1601 had at least one new SBI event. The incidence of stroke with focal neurological deficits was 3%. The prevalence of early post-procedural cognitive dysfunction (PCD) increased during follow-up, from 16% at 10.0±6.3 days to 26% at 6.1±1.7 months and meta-regression suggested an association between the mean number of new SBI and incidence of PCD. These data underline the importance of long-term follow-up, as several studies reveal a temporal association with cognitive dysfunction with mean follow-up times between 3.6 and 5.2 years, while those with short follow-up periods generally failed to detect any association^[48]^.

## Structural valve deterioration

The real clinical impact of structural valve deterioration (SVD) is still controversial, but it may be more frequent after TAVI than after SAVR. The lack of consensus definition for SVD across reports leads to conflicting results comparing the durability and longevity of the transcatheter valves and surgical bioprostheses^[49]^. Most of the patients included in these studies were at high surgical-risk, therefore with limited life expectancy, and received a first- or second-generation transcatheter heart valves (THV). Thus, in low-risk patients receiving third-generation THV the incidence of SVD remains undetermined.

Pibarot et al. sought to determine and compare the 5-year incidence of SVD, using a modified VARC-3 definition of SVD based on echocardiographic follow-up in intermediate-risk patients enrolled in the PARTNER 2A trial and registry, and in the SAPIEN 3 registry. In the PARTNER 2A trial, patients were randomly assigned to receive either TAVI with the SAPIEN XT or SAVR, whereas in the SAPIEN 3 registry, patients were assigned to TAVI with the SAPIEN 3. The study found an inferior durability of SAPIEN-XT versus the surgical heart valve with a 2.5-fold rate of SVD. Compared with SAVR, the SAPIEN-XT TAVI cohort had significantly higher 5-year incidence rates of SVD, SVD-related bioprosthetic valve failure (BVF), and all-cause (structural or nonstructural) BVF. On the other hand, the 5-year rates of SVD and SVD-related BVF in the SAPIEN 3 TAVI registry were not significantly different from a propensity score-matched SAVR cohort. A higher risk of all-cause BVF was observed with SAPIEN-3 compared to surgical valves, driven by more frequent valve reintervention in SAPIEN-3, mainly due to PVR^[49,50]^.

The results of the PARTNER 2A study showed a higher rate of reintervention (3.2% *vs*. 0.6%, *P*=0.003) and re-hospitalization (33.3% *vs*. 25.2%, *P*=0.006) related to the transcatheter valve compared to surgical prosthesis, in the 5 years after the initial intervention^[51]^.

## Prosthetic valve thrombosis

The incidence of subclinical prosthetic valve thrombosis after TAVI is still unclear but assumed more frequent after TAVI than after SAVR and may be a trigger for SVD. The prevalence of clinical valve thrombosis after TAVI has been reported around 1% (between 0.6%-2.8%), with a typically increased transprosthetic gradient associated with symptoms of heart failure and/or systemic thromboembolism^[52,53]^. However, subclinical leaflet thickening with reduced leaflet motion and manifested thrombosis in TAVI are more common than previously appreciated^[54-58]^.

Chakravarty et al.^[58]^ demonstrated that subclinical leaflet thrombosis occurs frequently in bioprosthetic aortic valves, more commonly in transcatheter than in surgical valves. The authors studied the prevalence of subclinical leaflet thrombosis in surgical and transcatheter aortic valves and the effect of novel oral anticoagulants (NOACs) in patients enrolled in the RESOLVE and SAVORY registries. Employing CT imaging with a dedicated four-dimensional volume-rendered imaging protocol, the subclinical leaflet thrombosis was found in 12% of the patients, 4% with thrombosis of surgical valves *versus* 13% of transcatheter valves. Subclinical leaflet thrombosis resolved in 100% of patients receiving anticoagulants (warfarin 67%, NOACs 33%), whereas it persisted in 91% of patients not receiving anticoagulants. Although stroke rates were not different between those with or without reduced leaflet motion, subclinical leaflet thrombosis was associated with increased rates of transient ischemic attacks and all strokes or TIAs^[58]^.

The OCEAN-TAVI registry, analyzing data from 485 patients who underwent post-TAVI 4-dimensional multidetector computed tomography to assess hypo-attenuated leaflet thickening with reduced leaflet motion compatible with thrombus at an average follow-up of 3 days, 6 months, 1 year, 2 years, and 3 years, showed that 9.3% of patients had early leaflet thrombosis on CT at a median of 3 days after TAVI, all subclinical. The rates of cumulative events of death, stroke, or readmission for heart failure over 2 years were 10.7% and 16.9% in patients with and without early leaflet thrombosis, respectively (P=0.63). Late leaflet thrombosis occurred up to 3 years, and male gender and PVR less than mild were independent predictors^[59]^.

The crimping and deployment of both balloon-expandable and self-expanding stent valves cause traumatic injury to the pericardial leaflets with collagen damage, predisposing to thrombus formation and accelerated calcification, a process even more accentuated if post-dilation is needed^[65]^. On the other hand, resection of the calcified native aortic valve leaflets during SAVR alters the flow dynamics after valve replacement compared with leaving native aortic valve cusps in situ during TAVI. Incomplete expansion or overexpansion of the transcatheter valves, compared with uniform expansion of the surgical valves, might alter mechanical stress on the leaflets, predisposing them to thrombus formation. Non-uniform expansion related to extensive calcifications is responsible for prosthetic device deformation that leads to an eccentricity > 10%, resulting in incomplete expansion of the metallic frame at almost all levels. In a fatigue simulation study, transcatheter valve leaflets were noted to sustain higher stresses, strains, and fatigue damage than did surgical aortic valve leaflets^[60]^. Finally, prosthetic valve thrombosis is considered a marker for SVD, and recent echocardiographic data from PARTNER-3 corroborated these concerns, observing leaflet thrombosis as the main cause of SVD at 1 year^[52]^.

The issue of whether routine anticoagulation would prevent leaflet thrombosis and improve clinical outcomes after TAVI was the focus of the GALILEO trial in which 1,644 patients undergoing TAVI were randomized to receive a rivaroxaban-based antithrombotic strategy (rivaroxaban plus aspirin) or an antiplatelet-based strategy (clopidogrel plus aspirin). While the rivaroxaban-based antithrombotic strategy was found more effective than an antiplatelet-based strategy in preventing subclinical leaflet motion abnormalities, the trial was terminated early owing to a higher rate of death or thromboembolic event and a higher rate of bleeding in the rivaroxaban group than in the antiplatelet group^[61,62]^.

Although the risk of post-TAVI cerebrovascular events peaks in the days following the procedure, this risk persists later, beyond the 30 days. Data suggest a potential relationship between subclinical thrombosis of the transcatheter prosthesis and cerebrovascular events (particularly transient ischemic attack)^[58]^. The PARTNER-3 echocardiographic findings agree with these concerns, observing leaflet thrombosis as the main cause of hemodynamic valve deterioration at 1 year^[52]^.

The PARTNER 3 RCT reported the 2-year clinical and echocardiographic outcomes comparing TAVI and SAVR in low-risk patients with a mean age of 73 years. A higher frequency of late valve thrombosis was found in TAVI patients at 24 months (2.6% *vs.* 0.7%; *P*=0.02), 63% of which presented between 1 and 2 years. However, the majority (75%) of patients were asymptomatic, and the diagnosis was largely driven by interval-mandated echocardiograms and subsequent computed tomography studies demonstrating hypoattenuated leaflet thickening and restricted leaflet motion. These findings were frequently associated with elevated mean valve gradients, lower effective orifice area and need for anticoagulation (with increased risk of bleeding) and disabling stroke in 2 patients^[18]^.

## Thrombocytopenia

Another emerging complication has been the incidence of TAVI-related thrombocytopenia, ranging in trials from 25% to 100%. Severe thrombocytopenia after TAVI was previously reported as a marker of early and late adverse outcomes, associated with worse clinical results, and identified as an independent risk factor for long-term mortality^[63-65]^. A >30% drop in platelet count values after TAVI was associated with higher rates of major bleeding and a risk of death within 30 days when compared to a drop ≤30%^[63]^. Balloon-expandable valves appear to induce more pronounced post-procedure thrombocytopenia than the self-expanding prosthesis^[66,67]^.

A range of hypotheses have been proposed to explain the post-TAVI platelet drop, but the underlying mechanisms for thrombocytopenia and post-TAVI mortality are not yet well understood and are considered multifactorial. The mechanism involves several components, with a sum of factors involving the device, the procedure, and the patient^[68]^.

## Infective endocarditis

The overall risk of prosthetic valve endocarditis (PVE) seems similar after SAVR and TAVI, the incidence is reported between 0.3-2.1 per 100 person-years, with younger patients, male, diabetes mellitus, and moderate to severe aortic regurgitation determining an increased risk.

Prosthetic valve endocarditis tends to occur relatively earlier in the post-TAVI period, with the average time from procedure to diagnosis varying from 5-12 months, and around 75% of cases occurring in the first year, in marked contrast to the historical SAVR series, where the incidence normally peaks from the second postoperative year onwards^[69]^.

Implantation of additional prosthetic material (either a second prosthesis to solve a periprocedural complication or valve-in-valve) may also be associated with an increased risk of subsequent PVE after TAVI. Remarkable is the deficient sensitivity of the echocardiogram in detecting PVE after TAVI, with characteristic vegetations seen in only 17-36% of cases in the initial investigation, highlighting the challenges of imaging in PVE after TAVI. The patient's prognosis is poor, with high in-hospital and long-term mortality. The in-hospital mortality ranges from 11-64% and the one-year mortality from 22-75%. Evidence is lacking to recommend surgical treatment in this group of patients^[69]^.

## Reoperation after TAVI

A recent study reporting the largest series of patients undergoing surgical reoperation after TAVI, using the Society of Thoracic Surgeons (STS) Adult Cardiac Surgery Database, involved 123 patients (median age 77 years) and reported higher than expected morbidity and mortality, associated with worse-than-expected outcomes as compared with similar patients initially receiving SAVR. The median time to reoperation was 2.5 months, and the operative mortality rate was 17.1%. Common indications for reoperation included early TAVI device failures such as paravalvular leak (15%), structural prosthetic deterioration (11%), failed repair (11%), sizing, or position issues (11%), and PVE (10%). All pre-operative risk categories were associated with an increased observed-to-expected mortality ratio^[70]^.

Although TAVI failure has been relatively rare, the absolute number is expected to rise and SAVR after failed TAVI will become more common, given the fact that TAVI volume is growing and the indication expanding to low-risk AS population. The management of TAVI structural deterioration occurring after 5 or more years will involve the management with SAVR somehow, as the role and outcomes of TAVI valve in valve in this setting are still undetermined.

The figures reported reflect the greater complexity with SAVR after TAVI, including longer operative and cardiopulmonary bypass times, some of patients requiring root replacement, and the surgical technical difficulties associated with the TAVI device removal, including the debridement of the supra- and sub-annular planes.

## Coronary occlusion

Coronary artery obstruction by valve debris released during the expansion of the TAVI prosthesis is a relatively infrequent complication, but has potentially catastrophic clinical consequences, with associated mortality of up to 50%. Coronary occlusion occurs in <1% of native valve interventions and tends to involve the left main coronary artery more frequently than the right coronary artery. Occlusion can also be caused by the displacement of the calcified leaflets. Coronary blocking causes a rapid worsening of the clinical and hemodynamic condition, with severe hypotension and dynamic changes in the ST segment and ventricular arrhythmias^[71]^.

## Vascular access complications

Currently, a significant reduction in major vascular complications after TAVI was driven by a combination of smaller sheath sizes, flexible delivery systems, prior assessment of peripheral vasculature by multidetector computed tomography, and operator experience. However, recent TAVI trials show an incidence of 6% to 8%, as seen in PARTNER-2 and SURTAVI, with vascular complications and bleeding remaining a significant challenge in contemporary practice and associated with longer hospital stays and higher mortality at 1 year^[71]^.

## Aortic root rupture

Aortic root rupture at the device landing zone is another occasional but serious complication after TAVI, with a reported incidence of 0.5% to 1%, with an overall mortality up to 48% and may reach 75% in cases of non-contained rupture, although the actual incidence can be greater when cases with protracted presentation are counted. Aortic ruptures are responsible for roughly 7% of all cases of conversion to an emergency surgery during TAVI. The most frequent anatomical site of rupture is the aortic annulus (involved in two-thirds of the cases), the left ventricular outflow tract is affected in 10%, sinus of Valsalva in 16%, and sinotubular junction rupture in 6%^[71]^.

## Alternative access - the transapical

The outcomes discussed above are mainly pertinent to TAVI via transfemoral access (TF). The alternative transapical access (TA-TAVI) has been progressively abandoned as a result of poorer outcomes, although still used when TF access is considered impracticable^[72]^. In the PARTNER I study, five-year mortality in high-risk patients favored SAVR compared to the TA-TAVI group, with survival curves continuously diverging over time^[73]^. The STACCATO randomized trial, which compared TA-TAVI with SAVR in low-risk elderly patients ≥75 years old, was prematurely discontinued because of excessive adverse events (death, stroke, acute renal failure, severe PVR) in the TA-TAVI group^[74]^.

## Volume-outcome ratio

TAVI trials in patients of moderate and low risk were conducted in high-volume centers with extensive experience in TAVI. Several reports have shown a strong relationship between hospital volume of TAVI procedures and patient outcomes, where the early benefits of TAVI over SAVR could be reduced in programs with less experience. Vemulapalli et al, analyzing the data from the Transcatheter Valve Therapy Registry concerning procedural volumes and outcomes, revealed a significant inverse association between the volume of TF-TAVI procedures and mortality from 2015 through 2017. Mortality at 30 days was higher and more variable at hospitals with a low procedural volume than at hospitals with a high procedural volume^[75]^.

## Appropriateness criteria

To put the results into perspective, the TAVI trials were never an all-comers enrollment of patients and the perspective of the exclusion criteria of the trials need to be made known. In general, patients with bicuspid aortic valves, aortic disease, the presence of significant calcification in the left ventricular outflow tract, complex coronary artery disease (with SYNTAX score> 22), and moderate or severe mitral and tricuspid insufficiency were excluded in most studies. For the PARTNER 3 study, 31 exclusion criteria were applied. Hence, the results cannot be generalized and broadly extended to the whole low-risk population.

## Costs

All of these aspects above stated affect the final cost of the procedure. In times of extreme pressure on health resources, regardless of the countries' economic position, the adoption of a new technology that is 5 to 10 times more expensive than the existing standard, with inferior results, requires serious reflection. Scrutinized through the Incremental Cost-Effectiveness Ratio (ICER), TAVI appears to represent an important financial burden even in the USA, the United Kingdom, and in European Union countries, and an unbearable burden for the economies of emerging countries^[76]^. While struggling to provide incremental resources in primary, secondary, and tertiary health care, adopting and expanding a much more expensive option with inferior outcomes seems illogical.

Many of the conclusions drawn early from RCT have gradually been contradicted by long-term follow-up of the patients and the analysis of registries and national databases with the inclusion of a large number of patients. However, as discussed in this article, there are many uncertainties about TAVI to patients broadly, but more worrying in the cohort of low/moderate surgical risk younger people. Only extended follow-up of patients will respond to these apprehensions and whether TAVI will remain competitive with surgery, or even with medical treatment, in the different subgroups of patients^[77]^.

## The controversy from the beginning - PARTNER studies

The publication of the results of the PARTNER trial - Pivotal Partner Trial - was decisive for the insertion and recommendations for TAVI in the American and European guidelines on management of valve diseases, making possible the rapid dissemination and acceptance of the treatment method worldwide. However, serious biases and controversies, and lack of data transparency coupled with serious conflicts of interest were insidious in these trials.

The PARTNER IB study set the role of TAVI in patients with severe AS who were not candidates for surgery, as TAVI led to an absolute 20% reduction in all-cause mortality in 1 year compared to “standard therapy”. The PARTNER cohort B trial is often quoted as the comparison between TAVI and medical treatment in inoperable patients. And it should have been, had it not been for the revelations that the authors modified the medical treatment cohort, by performing balloon aortic valvuloplasty (BAV) in 83% of patients in this control group. BAV was at that time and is now considered a class III recommendation when performed as a destination therapy in the treatment of severe AS.

Although providing an initial modest change in valve orifice area, with early symptomatic improvement, BAV induces serious acute and late complications, including acute severe aortic regurgitation (which makes the patient's condition more severe, since these patients have significant left ventricular hypertrophy with reduced left ventricular cavity), restenosis and clinical deterioration occurring within 6 to 12 months in most patients. In addition, the performance of BAV inflicted additional damage to patients in the medical treatment group, with 1.7% stroke in 30 days, vascular complications in 7.3%, and bleeding complications of vascular origin in 14%^[78-79]^.

Yet, compared to standard therapy, at 30 days, the TAVI group was associated with a higher incidence of stroke (5.0% *vs*. 1.7%, *P*=0.06) and major vascular complications (16.2 % *vs*. 1.1%, *P*<0.001). At 1 year, the death rate from any cause was 30.7% with TAVI compared to 50.7% with standard therapy (*P*<0.001). The rate of the composite outcome of death from any cause or repeated hospitalization was 42.5% with TAVI compared to 71.6% with standard therapy (*P*<0.001). Significant shortcomings in the trials remained obscured from the public domain and subsequent articles pointed out the unexpectedly high rate of deaths in the medical treatment arm related to the indiscriminate and harmful BAV utilization^[80,81]^. In addition, despite randomization, the treatment and control groups were unbalanced in a way that favored TAVI. The patients allocated to TAVI had a significantly better logistical EuroSCORE than those who received standard therapy (26.4±17.2 *vs*. 30.4±19.1, *P*=0.04). This difference raises the question of whether the best outcome (reduced rates of death from any cause) in patients who have undergone TAVI reflects the positive effect of experimental treatment or the best baseline conditions in this group of patients. The adjusted analysis would have produced a more realistic estimate of the effect size, which was not done.

Due to these problems, the FDA demanded a new trial, in which 41 inoperable patients were randomized to TAVI and 49 to medical therapy. Data presented at an FDA meeting on July 20, 2011, showed that patients in the TAVI arm had worse results than those who received medical therapy, with one-year mortality of 34.3% *versus* 21.6%, respectively. However, this new trial was never published^[82]^.

Relevant conflict of interest involved Martin B. Leon, the principal investigator for the PARTNER study, as revealed by several publications, who had substantial financial interests involved in the study. As the original developer of the Sapien valve, he received $ 6.9 million for the sale of the company he founded, Percutaneous Valve Technologies, for $ 125 million in 2004. Also, these publications reveal that he should receive three more payments for the achievement of three milestones: successful treatment of 50 patients, regulatory approval in Europe, and limited approval in the USA. In an interview with Businessweek, Leon said he had donated these millionaire payments to a school. But he refused to disclose the name of the school and has not made public the receipt^[80-82]^.

## The LACES statement

The Latin-American Association of Cardiac and Endovascular Surgery (LACES) issued a document disagreeing with the AHA/ACC guideline recommendations for selecting aortic valve procedures based on patients' age, as clinical trials thus far have not upfront assessed outcomes based on age range. Therefore, LACES considers an important methodological flaw subject to a high risk of inaccuracies to recommend as Class of Recommendation (COR) I, Level of Evidence A (greatest imprimatur of the guideline recommendations), an indication of TAVI or SAVR based on age. The age range used to recommend TAVI is well below the average age of trials in low-risk patients (73 years for PARTNER 3 and 74 years for EVOLUT Low Risk), therefore there is no reference to support this range defined by the AHA/ACC guideline authors. LACES reiterates that large RCT were built based on surgical risk. Since there is no evidence longer than a median of 5 years’ follow-up or recommendation over the safety of TAVI in patients of intermediate and low risk, LACES is not endorsing the recommendation for TAVI in patients with a life expectancy greater than 5 years.

According to LACES, no evidence shows TAVI superiority over SAVR at high surgical risk, the actual evidence being TAVI not inferior to SAVR, therefore the guideline recommendation should reflect this^[83]^.

## Information to patients

The evidence and data compiled reveal that patients with severe AS should be broadly and objectively informed about the available therapies, the risks involved, the benefits afforded, and the expected long-term prognosis. A shared decision-making should necessarily involve the patients and their family desire and preferences.

## The conflict of interest movement to restore credibility to evidence

At a time when public funding is limited for large RCTs, industry support is often essential to generate levels of evidence to answer important clinical questions. However, the involvement of the industry carries the risk of privileging the study design to obtain the desired result instead of pragmatic tests, interfering in protocol design, site selection and management, and in data analysis^[84]^. Comparative assessments sponsored by the industry systematically generate favorable results for sponsors, especially when the study design involves non-inferiority analysis^[10,85]^.

Investigating the association between industry funding and the statistical significance of results in published clinical and surgical trials, Bhandari et al. found that industry-funded trials are more likely to be associated with statistically significant pro-industry results, both in trials clinical and surgical interventions^[85]^. Ahn et al. investigated the association between the presence of individual principal investigators with financial ties to the manufacturer of the drug/device under study and the results of the study after accounting for the research funding source. They concluded that the financial ties of the principal investigators were independently associated with positive clinical trial results. These findings may suggest bias in the evidence base^[86]^. Dr. Marcia Angell, the former editor of the New England Journal of Medicine, in her commentary published in the Journal of the American Medical Association, stated that the results of recently published clinical research trials are often biased, habitually because the trials are designed to produce favorable results for the sponsor. This objective can be achieved in several ways, such as using "maneuvers that choose a composite outcome so that a favorable outcome can be selected as the “primary end outcome” or “minimizing the evidence of serious adverse effects”^[87]^.

But this gambit seems backfiring, as industry-sponsored trials are becoming increasingly discredited and under suspicion by the medical community, for being associated with flawed design and analysis. A renewed relationship has to be erected between the medical industry and the medical community to forge a new format of building trusted and reliable evidence. A worldwide movement is beginning to take shape over the substantial concerns that extensive financial conflicts of interest may unduly influence professional judgments, compromise the integrity of science, the objectivity of education, the quality of care, and public confidence in medicine. Led by the British Medical Journal (BMJ) in association with the Center for Evidence-Based Medicine at the University of Oxford, the global community of supporters of evidence-based medicine have mobilized to structure a manifesto for better evidence in medicine. The report recommended further research on conflicts of interest, improvements in transparency, and greater industry independence to strengthen confidence in the way evidence is produced and disseminated, and to drive more rational and safer use of medicines, devices, diagnostics, and data of public interest^[88]^.

These conflicts of interest and biases purportedly interfere with the appraisal and recommendation of the Heart Valve Team, where suppression and distortion of crucial information impacts a more balanced evaluation of a specific case and the best decision for patients. In this way, the AHA/ACC document, organized for the American reality and with the deficiencies outlined, misses the reliability and the purpose of serving as a reference for the world outside the United States.

## References

[1] Otto CM, Nishimura RA, Bonow RO, Carabello BA, Erwin 3rd JP, Gentile F (2021). 2020 ACC/AHA guideline for the management of patients with valvular heart disease: executive summary: a report of the American college of cardiology/American heart association joint committee on clinical practice guidelines. Circulation.

[2] Papanicolas I, Woskie LR, Jha AK (2018). Health Care Spending in the United States and Other High-Income Countries. JAMA.

[3] Tikkanen R, Abrams MK (2020). U.S. Health care from a global perspective, 2019: higher spending, worse outcomes?.

[4] (2017). The World Bank and WHO: Half the world lacks access to essential health services, 100 million still pushed into extreme poverty because of health expenses [Internet].

[5] Huygens SA, Etnel JRG, Hanif M, Bekkers JA, Bogers AJJC (2019). Bioprosthetic aortic valve replacement in elderly patients: Meta-analysis and microsimulation. J Thorac Cardiovasc Surg..

[6] Sharabiani MT, Fiorentino F, Angelini GD, Patel NN (2016). Long-term survival after surgical aortic valve replacement among patients over 65 years of age. Open Heart.

[7] Lassnigg A, Hiesmayr M, Frantal S, Brannath W, Mouhieddine M, Presterl E (2013). Long-term absolute and relative survival after aortic valve replacement: a prospective cohort study. Eur J Anaesthesiol.

[8] Kvidal P, Bergström R, Hörte LG, Ståhle E (2000). Observed and relative survival after aortic valve replacement. J Am Coll Cardiol.

[9] Baumgartner H, Iung B, Otto CM (2020). Timing of intervention in asymptomatic patients with valvular heart disease. Eur Heart J.

[10] Gaudino M, Ruel M, Obadia JF, De Bonis M, Puskas J, Biondi-Zoccai G (2021). Methodologic considerations on four cardiovascular interventions trials with contradictory results. Ann Thorac Surg.

[11] Barili F, Freemantle N, Parolari A (2020). Five-year outcomes with transcatheter aortic-valve replacement. N Engl J Med.

[12] Makkar RR, Thourani VH, Mack MJ, Kodali SK, Kapadia S, Webb JG (2020). Five-Year Outcomes of Transcatheter or Surgical Aortic-Valve Replacement. N Engl J Med.

[13] Barili F, Freemantle N, Pilozzi Casado A, Rinaldi M, Folliguet T, Musumeci F (2020). Mortality in trials on transcatheter aortic valve implantation versus surgical aortic valve replacement: a pooled meta-analysis of Kaplan-Meier-derived individual patient data. Eur J Cardiothorac Surg.

[14] Takagi H, Hari Y, Nakashima K, Kuno T, Ando T, All-Literature Investigation of Cardiovascular Evidence (ALICE) Group (2020). A meta-analysis of =5-year mortality after transcatheter versus surgical aortic valve replacement. J Cardiovasc Surg (Torino).

[15] Wang D, Huang L, Zhang Y, Cheng Z, Zhang X, Ren P (2020). Transcatheter aortic valve implantation versus surgical aortic valve replacement for treatment of severe aortic stenosis: comparison of results from randomized controlled trials and real-world data. Braz J Cardiovasc Surg.

[16] Tzamalis P, Alataki S, Bramlage P, Schmitt C, Schymik G (2020). Comparison of valve durability and outcomes of transcatheter aortic valve implantation versus surgical aortic valve replacement in patients with severe symptomatic aortic stenosis and less-than-high-risk for surgery. Am J Cardiol.

[17] Sayed A, Almotawally S, Wilson K, Munir M, Bendary A, Ramzy A (2021). Minimally invasive surgery versus transcatheter aortic valve replacement: a systematic review and meta-analysis. Open Heart.

[18] Leon MB, Mack MJ, Hahn RT, Thourani VH, Makkar R, Kodali SK (2021). Outcomes 2 Years After Transcatheter Aortic Valve Replacement in Patients at Low Surgical Risk. J Am Coll Cardiol.

[19] Sousa Uva M (2019). Transcatheter aortic valve implantation in low-risk patients: is it too early?. Heart.

[20] Kodali S, Pibarot P, Douglas PS, Williams M, Xu K, Thourani V (2015). Paravalvular regurgitation after transcatheter aortic valve replacement with the Edwards sapien valve in the PARTNER trial: characterizing patients and impact on outcomes. Eur Heart J.

[21] Leon MB, Smith CR, Mack MJ, Makkar RR, Svensson LG, Kodali SK (2016). Transcatheter or Surgical Aortic-Valve Replacement in Intermediate-Risk Patients. N Engl J Med.

[22] Popma JJ, Deeb GM, Yakubov SJ, Mumtaz M, Gada H, O’Hair D (2019). Transcatheter aortic-valve replacement with a self-expanding valve in low-risk patients. N Engl J Med.

[23] Ando T, Briasoulis A, Telila T, Afonso L, Grines CL, Takagi H (2018). Does mild paravalvular regurgitation post transcatheter aortic valve implantation affect survival? A meta-analysis. Catheter Cardiovasc Interv.

[24] Laakso T, Laine M, Moriyama N, Dahlbacka S, Airaksinen J, Virtanen M (2020). Impact of paravalvular regurgitation on the mid-term outcome after transcatheter and surgical aortic valve replacement. Eur J Cardiothorac Surg.

[25] Hamm CW, Möllmann H, Holzhey D, Beckmann A, Veit C, Figulla HR (2014). The German aortic valve registry (GARY): in-hospital outcome. Eur Heart J.

[26] Möllmann H, Kim WK, Kempfert J, Walther T, Hamm C (2015). Complications of transcatheter aortic valve implantation (TAVI): how to avoid and treat them. Heart.

[27] Faroux L, Chen S, Muntané-Carol G, Regueiro A, Philippon F, Sondergaard L (2020). Clinical impact of conduction disturbances in transcatheter aortic valve replacement recipients: a systematic review and meta-analysis. Eur Heart J.

[28] van Rosendael PJ, Delgado V, Bax JJ (2018). Pacemaker implantation rate after transcatheter aortic valve implantation with early and new-generation devices: a systematic review. Eur Heart J.

[29] Jarrett CM (2017). Permanent pacemaker insertion following transcatheter aortic valve replacement: not infrequent, not benign, and becoming predictable. J Thorac Cardiovasc Surg.

[30] Gaede L, Kim WK, Liebetrau C, Dörr O, Sperzel J, Blumenstein J (2018). Pacemaker implantation after TAVI: predictors of AV block persistence. Clin Res Cardiol.

[31] Mack MJ, Leon MB, Thourani VH, Makkar R, Kodali SK, Russo M (2019). Transcatheter Aortic-Valve Replacement with a Balloon-Expandable Valve in Low-Risk Patients. N Engl J Med.

[32] Feistritzer HJ (2020). SOLVETAV - a 2 x 2 randomized trial of self-expandable vs balloon-expandable valves and general vs local anesthesia in patients undergoing transcatheter aortic valve implantation: 1-year results [video].

[33] Mc Morrow R, Kriza C, Urbán P, Amenta V, Amaro JAB, Panidis D (2020). Assessing the safety and efficacy of TAVR compared to SAVR in low-to-intermediate surgical risk patients with aortic valve stenosis: an overview of reviews. Int J Cardiol.

[34] Rawasia WF, Usman MS, Mujeeb FA, Zafar M, Khan SU, Alkhouli M (2020). Transcatheter versus surgical aortic valve replacement in low-surgical-risk patients: a meta-analysis of randomized-controlled trials and propensity-matched studies. Cardiovasc Revasc Med.

[35] Muntané-Carol G, Urena M, Munoz-Garcia A, Padrón R, Gutiérrez E, Regueiro A (2020). Late cerebrovascular events following transcatheter aortic valve replacement. JACC Cardiovasc Interv.

[36] Messé SR, Mack MJ (2016). Improving outcomes from transcatheter aortic valve implantation: protecting the brain from the heart. JAMA.

[37] Haussig S, Mangner N, Dwyer MG, Lehmkuhl L, Lücke C, Woitek F (2016). Effect of a cerebral protection device on brain lesions following transcatheter aortic valve implantation in patients with severe aortic stenosis: the CLEAN-TAVI randomized clinical trial. JAMA.

[38] Gerckens U, Tamburino C, Bleiziffer S, Bosmans J, Wenaweser P, Brecker S (2017). Final 5-year clinical and echocardiographic results for treatment of severe aortic stenosis with a self-expanding bioprosthesis from the ADVANCE Study. Eur Heart J.

[39] Tay EL, Gurvitch R, Wijesinghe N, Nietlispach F, Wood D, Cheung A (2011). A high-risk period for cerebrovascular events exists after transcatheter aortic valve implantation. JACC Cardiovasc Interv.

[40] Hassell ME, Nijveldt R, Roos YB, Majoie CB, Hamon M, Piek JJ (2013). Silent cerebral infarcts associated with cardiac disease and procedures. Nat Rev Cardiol.

[41] Muralidharan A, Thiagarajan K, Van Ham R, Gleason TG, Mulukutla S, Schindler JT (2016). Meta-analysis of perioperative stroke and mortality in transcatheter aortic valve implantation. Am J Cardiol.

[42] Goldberg I, Auriel E, Russell D, Korczyn AD (2012). Microembolism, silent brain infarcts and dementia. J Neurol Sci.

[43] Ghanem A, Kocurek J, Sinning JM, Wagner M, Becker BV, Vogel M (2013). Cognitive trajectory after transcatheter aortic valve implantation. Circ Cardiovasc Interv.

[44] Roach GW, Kanchuger M, Mangano CM, Newman M, Nussmeier N, Wolman R (1996). Adverse cerebral outcomes after coronary bypass surgery. Multicenter study of perioperative ischemia research group and the ischemia research and education foundation investigators. N Engl J Med.

[45] Sacco RL, Kasner SE, Broderick JP, Caplan LR, Connors JJ, Culebras A (2013). An updated definition of stroke for the 21st century: a statement for healthcare professionals from the American heart association/American stroke association. Stroke.

[46] Windham BG, Deere B, Griswold ME, Wang W, Bezerra DC, Shibata D (2015). Small Brain Lesions and Incident Stroke and Mortality: A Cohort Study. Ann Intern Med.

[47] Pache G, Schoechlin S, Blanke P, Dorfs S, Jander N, Arepalli CD (2016). Early hypo-attenuated leaflet thickening in balloon-expandable transcatheter aortic heart valves. Eur Heart J.

[48] Woldendorp K, Indja B, Bannon PG, Fanning JP, Plunkett BT, Grieve SM (2021). Silent brain infarcts and early cognitive outcomes after transcatheter aortic valve implantation: a systematic review and meta-analysis. Eur Heart J.

[49] Van Belle E, Delhaye C, Vincent F (2020). Structural Valve Deterioration at 5 Years of TAVR Versus SAVR: Half Full or Half Empty?. J Am Coll Cardiol.

[50] Pibarot P, Ternacle J, Jaber WA, Salaun E, Dahou A, Asch FM (2020). Structural deterioration of transcatheter versus surgical aortic valve bioprostheses in the PARTNER-2 trial. J Am Coll Cardiol.

[51] Thourani VH, Kodali S, Makkar RR, Herrmann HC, Williams M, Babaliaros V (2016). Transcatheter aortic valve replacement versus surgical valve replacement in intermediate-risk patients: a propensity score analysis. Lancet.

[52] Pibarot P, Salaun E, Dahou A, Avenatti E, Guzzetti E, Annabi MS (2020). Echocardiographic results of transcatheter versus surgical aortic valve replacement in low-risk patients: the PARTNER 3 trial. Circulation.

[53] Faroux L, Alperi A, Muntané-Carol G, Rodes-Cabau J (2020). Safety and efficacy of repeat transcatheter aortic valve replacement for the treatment of transcatheter prosthesis dysfunction. Expert Rev Med Devices.

[54] Makkar RR, Fontana G, Jilaihawi H, Chakravarty T, Kofoed KF, De Backer O (2015). Possible Subclinical Leaflet Thrombosis in Bioprosthetic Aortic Valves. N Engl J Med.

[55] Hansson NC, Grove EL, Andersen HR, Leipsic J, Mathiassen ON, Jensen JM (2016). transcatheter aortic valve thrombosis: incidence, predisposing factors, and clinical implications. J Am Coll Cardiol..

[56] Vollema EM, Kong WKF, Katsanos S, Kamperidis V, van Rosendael PJ, van der Kley F (2017). Transcatheter aortic valve thrombosis: the relation between hypo-attenuated leaflet thickening, abnormal valve haemodynamics, and stroke. Eur Heart J.

[57] Fuchs A, De Backer O, Brooks M, de Knegt MC, Bieliauskas G, Yamamoto M (2017). Subclinical leaflet thickening and stent frame geometry in self-expanding transcatheter heart valves. EuroIntervention.

[58] Chakravarty T, Søndergaard L, Friedman J, De Backer O, Berman D, Kofoed KF (2017). Subclinical leaflet thrombosis in surgical and transcatheter bioprosthetic aortic valves: an observational study. Lancet.

[59] Yanagisawa R, Tanaka M, Yashima F, Arai T, Jinzaki M, Shimizu H (2019). Early and late leaflet thrombosis after transcatheter aortic valve replacement. Circ Cardiovasc Interv.

[60] Nappi F, Mazzocchi L, Timofeva I, Macron L, Morganti S, Avtaar Singh SS (2020). A Finite Element Analysis Study from 3D CT to Predict Transcatheter Heart Valve Thrombosis. Diagnostics (Basel).

[61] De Backer O, Dangas GD, Jilaihawi H, Leipsic JA, Terkelsen CJ, Makkar R (2020). Reduced Leaflet Motion after Transcatheter Aortic-Valve Replacement. N Engl J Med.

[62] Nishimura RA, Holmes DR Jr (2020). Treatment after TAVR - discordance and clinical implications. N Engl J Med.

[63] Jiritano F, Santarpino G, Serraino GF, Ten Cate H, Matteucci M, Fina D (2019). Peri-procedural thrombocytopenia after aortic bioprosthesis implant: a systematic review and meta-analysis comparison among conventional, stentless, rapid-deployment, and transcatheter valves. Int J Cardiol.

[64] Dvir D, Généreux P, Barbash IM, Kodali S, Ben-Dor I, Williams M (2014). Acquired thrombocytopenia after transcatheter aortic valve replacement: clinical correlates and association with outcomes. Eur Heart J.

[65] Mitrosz M, Kazimierczyk R, Chlabicz M, Sobkowicz B, Waszkiewicz E, Lisowska A (2018). Perioperative thrombocytopenia predicts poor outcome in patients undergoing transcatheter aortic valve implantation. Adv Med Sci.

[66] Hernández-Enríquez M, Chollet T, Bataille V, Campelo-Parada F, Boudou N, Bouisset F (2019). Comparison of the frequency of thrombocytopenia after transfemoral transcatheter aortic valve implantation between balloon-expandable and self-expanding valves. Am J Cardiol.

[67] Flaherty MP, Mohsen A, Moore 4th JB, Bartoli CR, Schneibel E, Rawasia W (2015). Predictors and clinical impact of pre-existing and acquired thrombocytopenia following transcatheter aortic valve replacement. Catheter Cardiovasc Interv.

[68] Gomes WJ (2019). Thrombocytopenia after aortic valve procedures - a possible not so harmless finding. Int J Cardiol.

[69] Allen CJ, Patterson T, Chehab O, Cahill T, Prendergast B, Redwood SR (2020). Incidence and outcomes of infective endocarditis following transcatheter aortic valve implantation. Expert Rev Cardiovasc Ther.

[70] Jawitz OK, Gulack BC, Grau-Sepulveda MV, Matsouaka RA, Mack MJ, Holmes DR Jr (2020). Reoperation after transcatheter aortic valve replacement: an analysis of the society of thoracic surgeons database. JACC Cardiovasc Interv.

[71] Scarsini R, De Maria GL, Joseph J, Fan L, Cahill TJ, Kotronias RA (2019). Impact of complications during transfemoral transcatheter aortic valve replacement: how can they be avoided and managed?. J Am Heart Assoc.

[72] Overtchouk P, Modine T (2018). Alternate access for TAVI: stay clear of the chest. Interv Cardiol.

[73] Mack MJ, Leon MB, Smith CR, Miller DC, Moses JW, Tuzcu EM (2015). 5-year outcomes of transcatheter aortic valve replacement or surgical aortic valve replacement for high surgical risk patients with aortic stenosis (PARTNER 1): a randomised controlled trial. Lancet.

[74] Nielsen HH, Klaaborg KE, Nissen H, Terp K, Mortensen PE, Kjeldsen BJ (2012). A prospective, randomised trial of transapical transcatheter aortic valve implantation vs. surgical aortic valve replacement in operable elderly patients with aortic stenosis: the STACCATO trial. EuroIntervention.

[75] Vemulapalli S, Carroll JD, Mack MJ, Li Z, Dai D, Kosinski AS (2019). Procedural volume and outcomes for transcatheter aortic-valve replacement. N Engl J Med.

[76] Manolis AS (2017). Transcatheter aortic valve implantation economics: a grisly reality. Ann Cardiothorac Surg.

[77] Hanzel GS, Gersh BJ (2020). Transcatheter aortic valve replacement in low-risk, young patients: natural expansion or cause for concern?. Circulation.

[78] Barbash IM, Waksman R (2012). Overview of the 2011 food and drug administration circulatory system devices panel of the medical devices advisory committee meeting on the Edwards SAPIEN™ transcatheter heart valve. Circulation.

[79] Redberg RF, Dhruva SS (2011). Transcatheter aortic-valve replacement. N Engl J Med.

[80] Van Brabandt H, Neyt M, Hulstaert F (2012). Transcatheter aortic valve implantation (TAVI): risky and costly. BMJ.

[81] (2006). Medicine in conflict [Internet].

[82] Meir B Doctor Is Pressed Again on Ties to Device Makers.

[83] Dayan V, Garcia-Villarreal OA, Escobar A, Ferrari J, Quintana E, Marin-Cuartas M (2021). The Latin American Association of Cardiac and Endovascular Surgery statement regarding the recently released 2020 ACC/AHA Guidelines for the Management of Patients with Valvular Heart Disease. Eur J Cardiothorac Surg.

[84] Flacco ME, Manzoli L, Boccia S, Capasso L, Aleksovska K, Rosso A (2015). Head-to-head randomized trials are mostly industry sponsored and almost always favor the industry sponsor. J Clin Epidemiol.

[85] Bhandari M, Busse JW, Jackowski D, Montori VM, Schünemann H, Sprague S (2004). Association between industry funding and statistically significant pro-industry findings in medical and surgical randomized trials. CMAJ.

[86] Ahn R, Woodbridge A, Abraham A, Saba S, Korenstein D, Madden E (2017). Financial ties of principal investigators and randomized controlled trial outcomes: cross sectional study. BMJ.

[87] Angell M (2008). Industry-sponsored clinical research: a broken system. JAMA.

[88] Moynihan R, Macdonald H, Heneghan C, Bero L, Godlee F (2019). Commercial interests, transparency, and independence: a call for submissions. BMJ.

